# Explaining the Varying Patterns of COVID-19 Deaths Across the United States: 2-Stage Time Series Clustering Framework

**DOI:** 10.2196/32164

**Published:** 2022-07-19

**Authors:** Fadel M Megahed, L Allison Jones-Farmer, Yinjiao Ma, Steven E Rigdon

**Affiliations:** 1 Farmer School of Business Miami University Oxford, OH United States; 2 Department of Epidemiology and Biostatistics College for Public Health and Social Justice Saint Louis University St Louis, MO United States

**Keywords:** explanatory modeling, multinomial regression, SARS-CoV-2, COVID-19, socioeconomic analyses, time series analysis

## Abstract

**Background:**

Socially vulnerable communities are at increased risk for adverse health outcomes during a pandemic. Although this association has been established for H1N1, Middle East respiratory syndrome (MERS), and COVID-19 outbreaks, understanding the factors influencing the outbreak pattern for different communities remains limited.

**Objective:**

Our 3 objectives are to determine how many distinct clusters of time series there are for COVID-19 deaths in 3108 contiguous counties in the United States, how the clusters are geographically distributed, and what factors influence the probability of cluster membership.

**Methods:**

We proposed a 2-stage data analytic framework that can account for different levels of temporal aggregation for the pandemic outcomes and community-level predictors. Specifically, we used time-series clustering to identify clusters with similar outcome patterns for the 3108 contiguous US counties. Multinomial logistic regression was used to explain the relationship between community-level predictors and cluster assignment. We analyzed county-level confirmed COVID-19 deaths from Sunday, March 1, 2020, to Saturday, February 27, 2021.

**Results:**

Four distinct patterns of deaths were observed across the contiguous US counties. The multinomial regression model correctly classified 1904 (61.25%) of the counties’ outbreak patterns/clusters.

**Conclusions:**

Our results provide evidence that county-level patterns of COVID-19 deaths are different and can be explained in part by social and political predictors.

## Introduction

**Figure 1:**
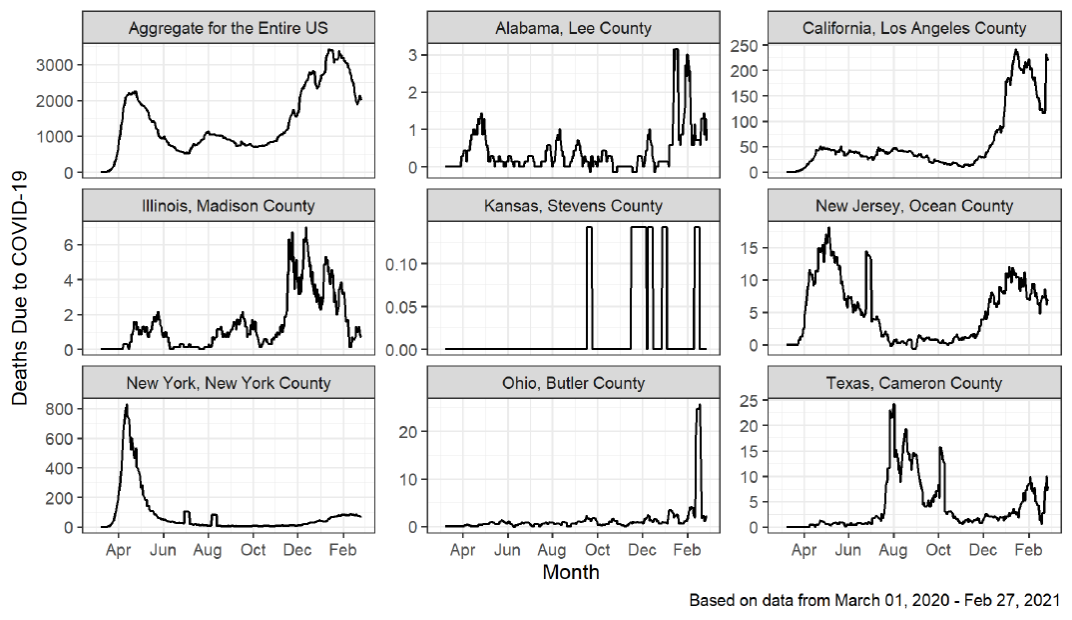
Time series profiles of the 7-day moving average of new COVID-19 deaths for the entire United States and 8 sample counties.

A geographically, politically, and socioeconomically diverse nation, the United States consists of 50 states, 48 of which are contiguous. When considering the COVID-19 pandemic in different regions throughout the United States, different patterns of outcomes emerge. Based on data obtained from the open source COVID-19 data hub [1], Figure 1 shows the national 7-day moving average of deaths as well as the various patterns that arise among 8 example counties from Sunday, March 1, 2020, to Saturday, February 27, 2021. For example, New York, NY, experienced a large first wave of deaths, followed by a relatively low death count through the remainder of the study. Nearby Ocean County, NJ, a populous county near the New Jersey shore had a large first wave of deaths, followed by a second wave beginning in late 2020. In contrast, Butler County, OH, a populous midwestern county, showed low death counts until late in the study period. None of these patterns mimics the overall pattern for the aggregate death counts in the United States.

Early in the COVID-19 pandemic, the county-level population mortality and case fatality rates were significantly different among the US regions [2]. Explanations for regional differences in health outcomes related to COVID-19 may be the structure of the government and policy making within the United States as it relates to the social vulnerability of the population. In the United States, each state consists of county governments that set health and economic policies for local communities. The counties within the states vary in terms of population size, demographics, access to health care, housing, and transportation. Some have noted that the regional differences in COVID-19 policies, compliance, and subsequent outcomes could be due to political differences across the regions. Goldwitzer et al [3] showed Republican-leaning counties displayed less physical distancing compared to Democratic-leaning counties and a subsequent increase in COVID-19 cases and deaths. Another study showed Democratic governors were 50% more likely to implement stay-at-home orders [4], which have been associated with increased physical distancing and reduction in COVID-19 cases and deaths [5].

Here, we investigate the regional patterns in deaths attributed to COVID-19. The phenomenon of differing national and regional patterns within the United States was illustrated for confirmed COVID-19 cases in Megahed et al [6]. In addition, a report by the *Financial Times* [7] argued, “Across the world, public health data are gathered at a very local level before aggregation into regional and national figures.... While useful as a summary, local distinctions get lost, painting a misleading image of whole countries being affected uniformly.” In this study, we investigated the various patterns of COVID-19 deaths across 3108 contiguous counties in the United States. We also sought to determine what factors relate to the pattern of deaths. Specifically, we posed 3 questions:

- How many distinct clusters of counties in the United States exhibit similar time series patterns in the deaths due to COVID-19?
- How are these clusters geographically distributed across the United States?
- Are certain geographic, political, government, and social vulnerability variables associated with the patterns of COVID-19 related deaths?

To address the first question, we performed a cluster analysis on the time series of the 3108 US counties. We provided maps to show the geographic distribution of the clusters. To address the third question, we applied a multinomial logistic regression analysis using geographic, political, and social vulnerability data to explain the patterns of deaths due to COVID-19 over time.

## Methods

This study was conducted in 3 stages: (1) data gathering and preprocessing, (2) time series clustering, and (3) modeling and cluster validation.

### Data

The open source COVID-19 data hub [1] was used to extract county-level time series data related to confirmed COVID-19 deaths from Sunday, March 1, 2020, to Saturday, February 27, 2021. Data were extracted from 3108 counties in the 48 contiguous US states and were completely anonymous. This data set was used to compute the daily confirmed deaths related to COVID-19 by county and contained the sole data used to inform the time series cluster analysis.

To develop the explanatory model describing the clusters, the following additional variables were gathered: region, governor's party affiliation, government response. the Centers for Disease Control and Prevention’s (CDC) social vulnerability index (SVI), and population density.

#### Region

**Figure 2:**
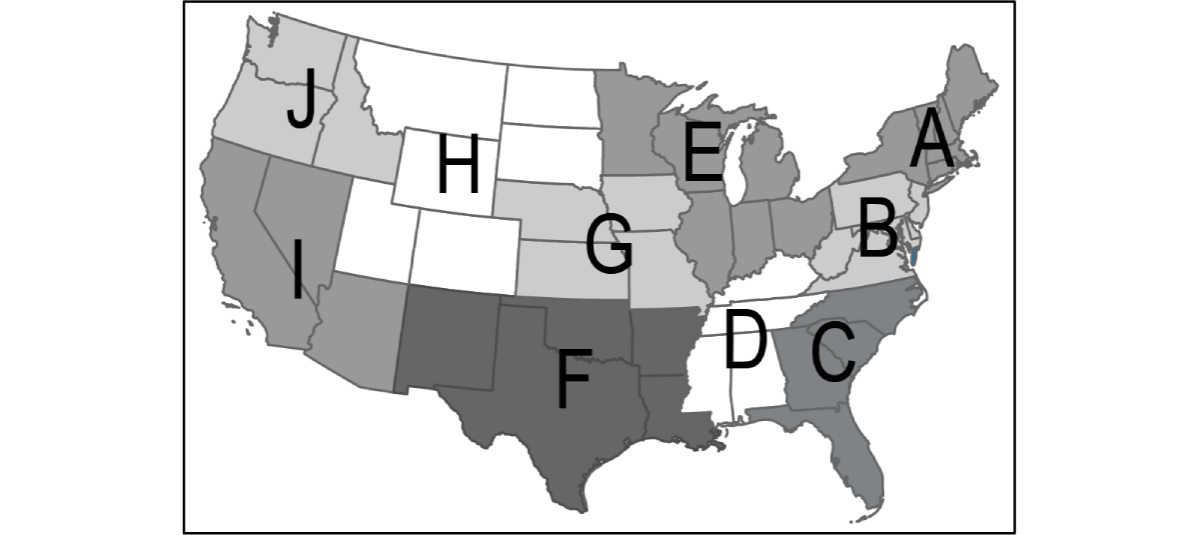
The 10 CDC regions. CDC: Centers for Disease Control and Prevention.

The CDC produces a 10-region Framework for Chronic Disease Prevention and Health Promotion [8]. Figure 2 shows the 10 regions used in our explanatory model. The CDC's National Center for Chronic Disease Prevention and Health Promotion (NCCDPHP) developed these regions to promote consistency in technical assistance and communications for chronic disease prevention [8].

#### Governor's Party Affiliation

The political party affiliation of each US state governor (within the 48 contiguous US states) at the start of the pandemic (March 2020) was determined. Since the District of Columbia does not have a governor, the political party of the mayor (Democrat) was used. The party affiliation of the governor was used as this affects the political actions and policies taken, often in the form of executive orders from the governor, during the pandemic [4].

#### Government Response

The overall government response index (at the US state level) from the Blavatnik School of Government [9] was downloaded on March 16, 2021. The index considers containment and closure indicators, such as school and workplace closings; economic response, such as income support and debt relief; and health systems, such as testing policies, contact tracing, and investment in vaccines. Higher values of the government response index indicate a stronger government response related to the pandemic. This index changed over the time of the study period. To capture the index over the majority of the study period, we summarized the index using the median value over the study period. Details of the methodology used to compute the index can be found at Oxford University COVID-19 Tracker Github [10].

#### The Social Vulnerability Index

The CDC’s SVI is computed by the CDC's Agency for Toxic and Disease Registry's Geospatial Research, Analysis, and Services Program [11]. The SVI provides the relative vulnerability of each US county based on US Census data and is ranked on 15 social factors, including unemployment, minority status, and disability. Note that the SVI data from the CDC returned results for 3107 counties, with no data on Rio Arriba County, New Mexico, and hence this county was excluded from our explanatory analysis. The SVI data were grouped into the following 4 themes:

- SVI theme 1: socioeconomic
- SVI theme 2: household composition and disability
- SVI theme 3: minority status and language
- SVI theme 4: housing and transportation

Our study included each of the 4 SVI themes. To construct the SVI for each theme, the percentile rank for each variable across the counties was computed. These were summed across the themes and then ranked within each domain. The SVIs ranged from 0 to 1, with higher values of SVIs for a particular theme indicating a higher level of social vulnerability. For more details on the SVI, see Flanagan et al [12].

#### Population Density

The population density in each county was computed based on the land area in square miles and the 2014-2018 American Community Survey (ACS) population estimates in each county. Both land area and population estimate variables were obtained from the CDC’s SVI 2018 data set [11]. Due to right-skewness in this variable, the natural logarithm of population density was used in the analysis.

### Time Series Clustering

Time series cluster analysis was based solely on the daily confirmed deaths related to COVID-19 by county. The goal was to separate counties into groups (clusters) that show similar time series patterns. There are 3 important decisions that affect the cluster solution: (1) the scaling of the data, (2) the measure of distance between the clusters, and (3) the clustering algorithm. Liao [13] gives an overview of time series clustering methods.

For this study, the daily confirmed deaths related to COVID-19 by county were smoothed using a 7-day moving average to account for weekly patterns due to reporting. Moreover, the 7-day moving averages were rescaled so that all values fell between 0 and 1 to focus on the pattern of the progression of the deaths rather than the magnitude of the death counts. The magnitude of the death counts in each county depends on many factors, such as county size, population density, and region. The scaled 7-day moving average for county i at time t is




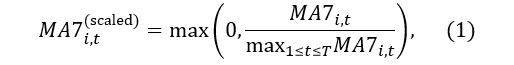




where *MA7_i,t_* is the 7-day moving average of deaths related to COVID-19 for county *i* at time *t*. The maximum in the denominator is taken over all time, *0≤t≤T*. The outer maximum function in Equation (1) is used to account for reporting adjustments that occur with negative death counts on some days.

For illustration, suppose that county *i* recorded deaths only on days 7, 8, and 9, when, respectively, 7, 21, and 14 deaths occurred. On all other days, no deaths were recorded. For clarity, this sequence of death counts, the calculations of the 7-day moving averages (*MA7_i,t_*), and the scaled moving averages (
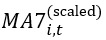
) for the first 17 days are shown in Table 1.

This method of scaling the 7-day moving averages ensured that we evaluated the shape of the death profile for each county across time.

Many metrics can be used to measure the distance between time series, including Euclidean distance, dynamic time warping [14], and the Pearson correlation coefficient. An elastic measure, such as dynamic time warping, is commonly used with time series clustering [13] because it aligns or *warps* the time series so that the distance between them is minimized. Elastic measures such as this do not preserve the timing of the outbreak and deaths in a meaningful way. For this reason, we used the Euclidean distance to measure the distance between the time series clusters. In our case, the Euclidean distance between 2 death profiles of length *T* was




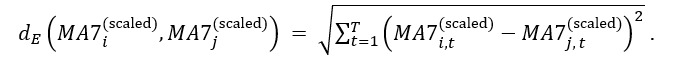




There are numerous clustering algorithms that have been suggested for time series clustering [13,15]. We used *k*-means clustering for this analysis. A heuristic-based method of clustering, *k*-means clustering partitions *n* objects into *k≤n* mutually exclusive clusters and each cluster is represented by the most centrally located object in the cluster. One limitation of the *k*-means clustering approach is that the number of clusters must be determined a priori in order to obtain a solution. It is common practice in exploratory research to evaluate cluster solutions for several sizes of *k* and select the *best* based on measures of cluster validity or homogeneity [16]. The R package *NbClust* [17] can be used to compute up to 30 cluster validity indices for cluster solutions of several sizes, *k*. This approach provides a systematic, data-driven method for selecting the optimal number of clusters in a data set without capitalizing on a single validity measure. For this analysis, *k*-means clustering was used to find the cluster solutions and the *NBClust* package was used to determine the optimal number of clusters to retain.

**Table 1:** Example calculation of the scaled 7-day moving averages (
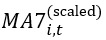
).

Time	1	2	3	4	5	6	7	8	9	10	11	12	13	14	15	16	17
Deaths	0	0	0	0	0	0	7	21	14	0	0	0	0	0	0	0	0
*MA7_i,t_*	N/A^a^	N/A	N/A	N/A	N/A	N/A	1	4	6	6	6	6	6	5	2	0	0
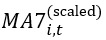	N/A	N/A	N/A	N/A	N/A	N/A	1/6	4/6	1	1	1	1	1	5/6	2/6	0	0

^a^N/A: not applicable.

### Explanatory Modeling

The time series clustering method described before resulted in mutually exclusive clusters of time series profiles containing counties with similar patterns in the daily deaths related to COVID-19. To further validate the cluster solution and to explain the differences in the progression of daily deaths across the counties, a multinomial regression analysis [18] was fit using the explanatory variables described in the Data section. The *multinom* function from the R package *nnet* [19] was used for this analysis.

Model performance was evaluated in terms of the ability to meaningfully interpret the model coefficients and by evaluating the in-sample classification performance. Specifically, the model predicted cluster was compared to the cluster as determined by the time series cluster solution for each county. The in-sample classification performance was measured by sensitivity, specificity, and balanced accuracy:

Sensitivity = TP/(TP + FN),

where TP and FN are the number of true-positive and false-negative predictions, respectively,

Specificity = TN/(TN + FP),

where TN and FP are the number of true-negative and false-positive prediction, respectively, and

Balanced accuracy = (Sensitivity + Specificity)/2.

## Results

### Number of Distinct Clusters

**Figure 3:**
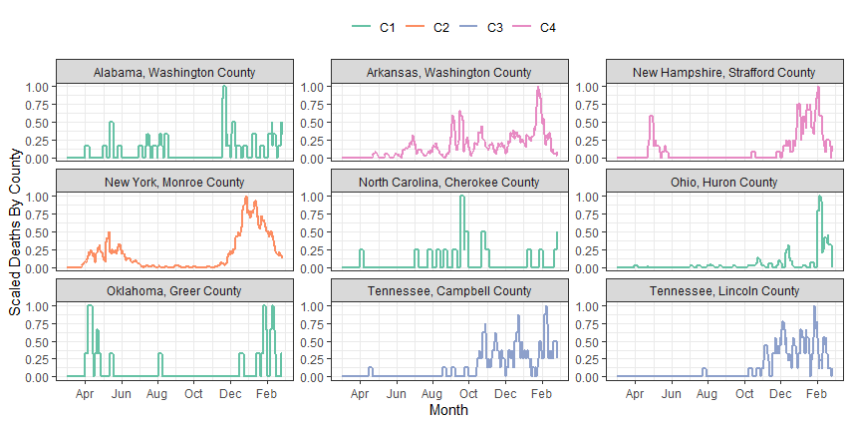
Time series profiles of the scaled 7-day moving average of new COVID-19 deaths for 9 sample counties.

To address our first research question regarding the number of distinct clusters, we used time series cluster analysis of the scaled 7-day moving average of daily deaths due to COVID-19. Figure 3 shows the scaled time series of the daily deaths due to COVID-19 for 9 randomly selected contiguous counties in the United States during the study period. We evaluated 2≤*k*≤51 time series cluster solutions using 23 cluster validity indices [17]. Of the 23 validity indices, 7 (30.4%) preferred a 4-cluster solution. The second-most preferred cluster solution was a 2-cluster solution, which was preferred by 6 (26.1%) of the 23 indices. Using a majority rule of the validity indices, we retained a 4-cluster solution.

**Figure 4:**
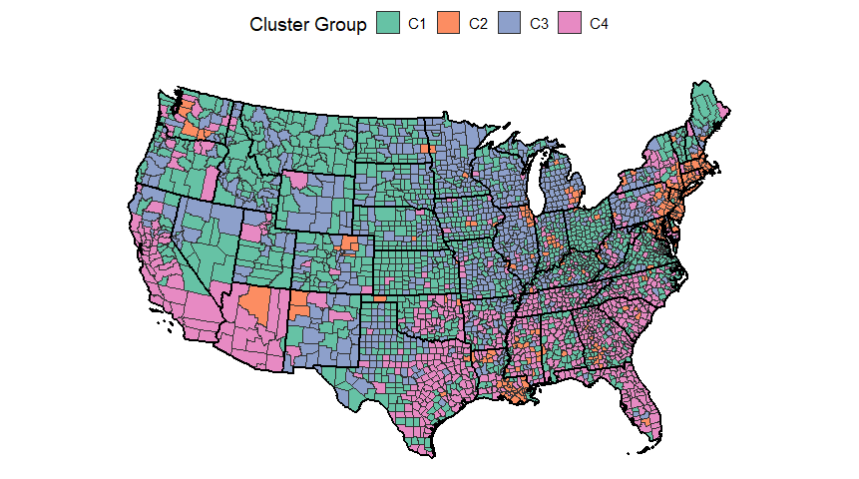
Map of 4 scaled time series profile clusters of COVID-19 deaths by county in contiguous US counties.

Figure 4 shows the geographic distribution of the 4-cluster solution across the United States. Cluster C1 is primarily concentrated in the Upper Midwest and mountain states, as well as in Ohio, Central Kentucky, Virginia, and Maine. Cluster C2 is located along the coast in the Northeast and in some of the larger US cities, such as Chicago, Detroit, Seattle, and New Orleans. Cluster C3 is scattered throughout much of the United States but particularly in Missouri, Illinois, and the states surrounding the Great Lakes. Cluster C4 occurs across the United States but shows concentrations in California, East Texas, the Southwest, and the Southeast. For an interactive color version of this map, please see Section 3.3.3 in Megahed et al [20].

**Figure 5:**
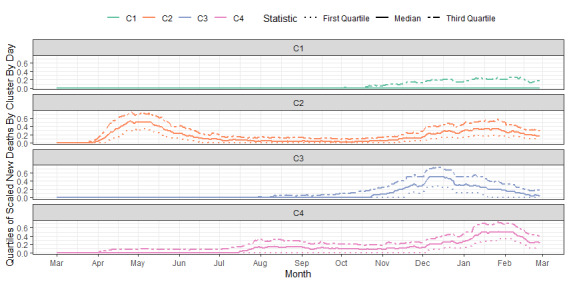
A summary plot, where the median scaled time series profile for each cluster is depicted using the solid bold line. The first and third quartiles are shown by dotted and 2-dash lines, respectively.

Figure 5 shows the 25^th^, 50^th^, and 75^th^ percentiles of the time series profiles for the counties within each cluster and provides insight into the shape of the cluster patterns. From Figure 5, it is clear that counties in cluster C1 experienced a low number of deaths due to COVID-19 throughout the study period. Counties clustering in C2 experienced early death counts beginning in April 2020, but the death counts tapered off in early summer. These counties maintained low death counts throughout the late summer and early fall, until rising again in November 2020. In C3, counties experienced few COVID-19 deaths until October 2020, when they saw a rapid rise in deaths. The death counts in C3 began dropping in December 2020, which continued through March 2021. The fourth cluster, C4, showed a small increase in deaths in late summer, followed by a steady rise throughout the fall and a higher peak in early 2021.

### Explaining the Clusters

To address the second research question regarding factors that relate to the patterns of COVID-19–related deaths, we used an explanatory multinomial regression analysis to validate our cluster solution. Table 2 provides a summary of the explanatory study variables for each cluster.

Table 3 gives the coefficients from the multinomial logistic regression analysis. The dependent variable was cluster. The baseline category for the analysis was C1, the cluster of counties with few deaths related to COVID-19. The coefficients showed the linear change in the natural log of the odds ratio (OR) of a county classified in a corresponding cluster (eg, C2, C3, or C4) versus the baseline cluster (C1). From Table 3, it is clear that several geographic, political, government, and social vulnerability variables are associated with the patterns in COVID-19–related deaths.

**Table 2:** A summary of how the predictor variables were distributed per cluster. For each numeric variable, we report the mean (SD). For categorical variables, we report the distribution of each subcategory across the 4 clusters. The row summation of percentages for a subcategory may deviate slightly from 100% due to rounding errors.

Variables		C1 (N=1261)		C2 (N=226)		C3 (N=827)		C4^a^ (N=794)		
**Continuous variables, mean (SD)**										
	Theme 1: socioeconomic	0.48 (0.30)	0.44 (0.31)		0.45 (0.27)		0.61 (0.26)			
	Theme 2: household composition and disability	0.50 (0.28)	0.37 (0.31)		0.49 (0.28)		0.56 (0.29)			
	Theme 3: minority status and language	0.41 (0.28)	0.71 (0.22)		0.43 (0.27)		0.65 (0.24)			
	Theme 4: housing and transportation	0.42 (0.29)	0.60 (0.28)		0.49 (0.26)		0.60 (0.27)			
	Log(population density)	3.01 (1.71)	5.86 (1.81)		3.73 (1.31)		4.60 (1.29)			
	Government response index median	47.09 (8.45)	52.87 (9.13)		47.24 (8.25)		48.13 (7.65)			
**Categorical variables, n (%)**										
	Governor’s party (Democratic)	579 (45.9)	142 (62.8)		428 (51.8)		202 (25.4)			
	Governor’s party (Republican)	682 (54.1)	84 (37.2)		399 (48.2)		591 (74.4)			
	Region A	41 (3.3)	43 (19.0)		21 (2.5)		24 (3.0)			
	Region B	131 (10.4)	63 (27.9)		62 (7.5)		48 (6.0)			
	Region C	101 (8.0)	19 (8.4)		13 (1.6)		239 (30.1)			
	Region D	140 (11.1)	20 (8.8)		51 (6.2)		153 (19.3)			
	Region E	188 (14.9)	30 (13.3)		283 (34.2)		23 (2.9)			
	Region F	154 (12.2)	31 (13.7)		116 (14.0)		201 (25.3)			
	Region G	236 (18.7)	7 (3.1)		144 (17.4)		25 (3.1)			
	Region H	187 (14.8)	7 (3.1)		88 (10.6)		9 (1.1)			
	Region I	22 (1.7)	1 (0.4)		14 (1.7)		53 (6.7)			
	Region J	61 (4.8)	5 (2.2)		35 (4.2)		18 (2.3)			

^a^Rio Arriba County, New Mexico, assigned to C4 based on the time series clustering was not modeled using the multinomial logistic regression, since we could not obtain values for its predictor variables. Hence, the reported mean (SDs) and n (%) for C4 exclude this county.

**Table 3:** Results of multinomial logistic regression for clusters C2, C3, and C4. We used C1 as the reference cluster since it contained the largest number of counties.

Variables	C2		C3			C4	
	β (SE)	OR^a^ (95% CI)	β (SE)	OR (95% CI)	β (SE)		OR (95% CI)
Theme 1: socioeconomic	0.419 (0.592)	1.52 (0.48-4.85)	–0.356 (0.286)	0.70 (0.40-1.23)	–0.018 (0.376)		0.98 (0.47-2.05)
Theme 2: household composition and disability	–0.245 (0.432)	0.78 (0.34-1.83)	0.392 (0.223)	1.48 (0.96-2.29)	0.638 (0.267)		1.89 (1.12-3.19)
Theme 3: minority status and language	3.661 (0.469)	38.90 (15.51-97.54)	0.004 (0.222)	1.00 (0.65-1.55)	1.162 (0.268)		3.20 (1.89-5.40)
Theme 4: housing and transportation	0.557 (0.428)	1.75 (0.75-4.04)	1.086 (0.227)	2.96 (1.90-4.62)	0.599 (0.270)		1.82 (1.07-3.09)
Log(population density)	1.009 (0.078)	2.74 (2.35-3.20)	0.417 (0.043)	1.52 (1.39-1.65)	0.959 (0.057)		2.61 (2.33-2.92)
Governor’s party (Republican)	–0.101 (0.233)	0.90 (0.57-1.43)	–0.323 (0.122)	0.72 (0.57-0.92)	1.093 (0.173)		2.98 (2.13-4.19)
Region B	–1.879 (0.464)	0.15 (0.06-0.38)	–0.509 (0.354)	0.60 (0.30-1.20)	–1.108 (0.395)		0.33 (0.15-0.72)
Region C	–2.621 (0.496)	0.07 (0.03-0.19)	–1.673 (0.437)	0.19 (0.08-0.44)	0.502 (0.376)		1.65 (0.79-3.45)
Region D	–1.717 (0.537)	0.18 (0.06-0.51)	–0.574 (0.369)	0.56 (0.27-1.16)	0.242 (0.401)		1.27 (0.58-2.80)
Region E	–1.941 (0.461)	0.14 (0.06-0.35)	0.884 (0.324)	2.42 (1.28-4.57)	–1.925 (0.403)		0.15 (0.07-0.32)
Region F	–1.520 (0.522)	0.22 (0.08-0.61)	0.629 (0.367)	1.88 (0.91-3.85)	0.814 (0.444)		2.26 (0.95-5.39)
Region G	–2.886 (0.647)	0.06 (0.02-0.20)	0.363 (0.361)	1.44 (0.71-2.92)	–1.536 (0.444)		0.22 (0.09-0.51)
Region H	–2.221 (0.681)	0.11 (0.03-0.41)	0.374 (0.396)	1.45 (0.67-3.16)	–1.329 (0.570)		0.26 (0.09-0.81)
Region I	–3.509 (1.117)	0.03 (0.00-0.27)	0.657 (0.479)	1.93 (0.75-4.93)	2.139 (0.476)		8.49 (3.34-21.58)
Region J	–2.527 (0.666)	0.08 (0.02-0.29)	0.228 (0.396)	1.26 (0.58-2.73)	–0.213 (0.480)		0.81 (0.32-2.07)
Government response	–0.028 (0.018)	0.97 (0.94-1.01)	–0.030 (0.009)	0.97 (0.95-0.99)	–0.020 (0.012)		0.98 (0.96-1.00)
Constant	–5.171 (1.292)	0.01 (0.00-0.07)	–1.308 (0.684)	0.35 (0.09-1.35)	–5.115 (0.934)		0.01 (0.00-0.04)

^a^OR: odds ratio.

We found that the clusters can be roughly described as follows:

- C1: low death rates throughout much of the pandemic; found mostly in Upper Midwest and mountain states
- C2: high death rates in spring 2020, with another spike in December 2020/January 2021; found mostly in the northeast and other large cities
- C3: low death rates until fall 2020, followed by a peak in December 2020; spread throughout the United States with concentrations in Central Midwest and Great Lakes
- C4: steady death rates from late summer through December 2020, followed by a peak in January; spread throughout the United States with concentrations in California, the Southwest, and the Southeast

“SVI theme 3: minority status and language” was significantly associated with clustering in C2 versus C1, yielding an OR of 38.90. Counties with high levels of SVI theme 3 were strongly associated with membership in C2 compared to C1. All CDC regions (B-J) showed a significant, negative association with C2 versus C1, indicating that being located outside region A (the Northeast, baseline category for region) is associated with lower odds of clustering in C2 versus C1. This is consistent with our initial finding from the map in Figure 4, which showed that the counties in C2 are primarily located in the Northeast.

The variable with the strongest positive association to C3, relative to C1, was “SVI theme 4: housing and transportation.” Population density was also significant and positively related to C3. The governor's party was significant and negatively associated with C3, indicating that counties in states with Republican governors are associated with lower odds of clustering in C3 than in C1. The government response was also significant and negatively related to membership in C3, but the effect was small. Among the regions, the coefficient for region C (North Carolina, South Carolina, Georgia, and Florida) was significant and negative; thus, counties in these states are associated with lower odds of being classified in C3 than in C1. In contrast, the coefficient for region E was significant and positive, which suggests that counties in Minnesota, Wisconsin, Illinois, Indiana, Michigan, and Ohio are associated with higher odds of clustering in C3.

“SVI theme 1: socioeconomic” was not significant for membership in any of clusters C2-C4; however, 3 of the SVIs (household composition and disability, minority status and language, and housing and transportation) were significant and positively associated with membership in C4. In addition, counties located in states with Republican governors were also associated with higher odds of classification in C4 relative to C1. Among the CDC regions, regions I (California, Nevada, and Arizona) and F (New Mexico, Texas, Oklahoma, and Louisiana) had positive coefficients. Regions B, E, G, and H had significantly negative coefficients. The logarithm of population density was also a significant predictor for classification in C2, C3, and C4, relative to C1, which indicates that a low population density is associated with clustering in C1.

**Figure 6:**
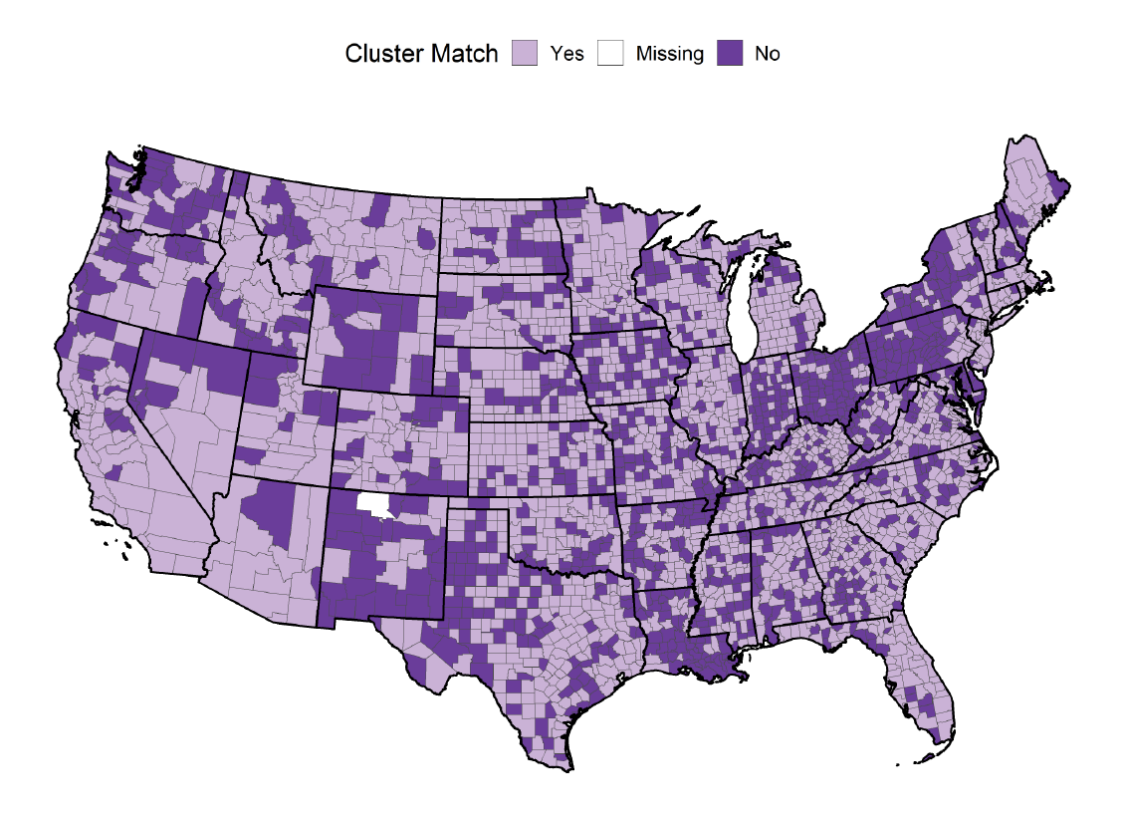
Map of the prediction accuracy of the multinomial logistic model describing the time series cluster solution. Counties in a light color (labeled “Yes”) were correctly classified by the model. Counties in a dark color (labeled “No”) were incorrectly classified. Rio Arriba County, New Mexico (in white), was not classified due to missing data.

Overall, the multinomial regression model correctly classified 1904 (61.25%) of the 3108 counties into 1 of 4 clusters. Table 4 gives the in-sample predictive performance of the multinomial regression model broken down by cluster. The balanced accuracy was similar for all 4 clusters, ranging from 0.63 to 0.80. A more nuanced view of the performance can be seen from sensitivity and specificity. The model performed well in correctly classifying counties in cluster C4 (sensitivity=0.74), which shows a sustained emergence in deaths beginning in late summer 2020. The model also performed well in classifying counties in cluster C1 (sensitivity=0.71), counties with few deaths. However, it had only moderate ability to correctly classify counties into clusters C2 and C3 (sensitivity=0.42 and 0.39, respectively). Note that the sensitivity performance for clusters C2 and C3 exceeded the expected sensitivity of 0.25 that would be obtained from random allocation among 4 classes in a balanced or imbalanced multiclass classification problem (see Megahed et al [21] for more details). In terms of specificity, the model performed well at identifying which counties are not in clusters C1-C4, with specificity values ranging from 0.71 to 0.98. Figure 6 shows the distribution of the accuracy of the multinomial logistic model in predicting cluster membership. Counties that were correctly predicted from the model are indicated in a light color, while those that were incorrectly predicted are indicated in a dark color. The model provides some insight into the patterns across the United States, but additional data are needed to more accurately classify counties in terms of the pattern of death rates due to COVID-19. For an interactive version of this map, please see Section 4.2.4 in Megahed et al [20].

**Table 4:** The predictive performance of the multinomial regression model for each cluster.

Cluster	Balanced accuracy	Sensitivity	Specificity
C1	0.71	0.71	0.71
C2	0.70	0.42	0.98
C3	0.63	0.39	0.88
C4	0.80	0.74	0.86

## Discussion

### Principal Findings

This research provides a framework for understanding the pattern of COVID-19–related deaths across the United States. Using time series clustering with county-level data on the occurrence of COVID-19–related deaths, we observed 4 distinct patterns from March 1, 2020, to February 27, 2021. The second stage of our analysis revealed that these patterns can be partially explained by region as well as social and political predictors.

Our findings add to the literature on the relationship between COVID-19 outcomes and vulnerable populations [22-24]. The largest number of counties in the United States experienced few deaths during the study period (cluster C1). These counties were, on average, at or below the median of all measures of social vulnerability. With lower population densities, and spread throughout the United States, C1 counties served as our model baseline.

The county-level COVID-19 death data were extracted using the COVID19 R package [1], which extracted confirmed deaths from a GitHub repository [25]. The cross-sectional data set containing the predictors used in the multinomial regression was compiled by the authors from disparate sources and is available in Megahed [26]. R statistical software version 4.0.4 was used for all processing and analysis of data. A reproducible workflow of our analysis is made available using R Markdown and is hosted in Megahed et al [20], following the best practices of Jalali et al [27] in reporting and documenting analyses for COVID-19.

Cluster C3 (low death rates until fall 2020, peaking in December 2020) had the second largest number of counties. C3 counties are spread across much of the country but have concentrations in the Great Lakes and Central Midwest regions. Interestingly, few incidences of C3 occur in the Southeastern United States and along the eastern seaboard from Washington, DC, to Massachusetts. Like C1, counties in C3 had SVI measures below the median, on average. These counties experienced a single late wave in COVID-19 deaths beginning in late October 2020 that declined by the end of the study period. There were a few distinguishing features between counties being classified in C3 versus C1: a higher population density, Democratic state leadership, location outside the Southeast, location in the Great Lakes region, and higher vulnerability in the SVI housing and transportation theme. This index indicates a higher incidence of multiunit housing, mobile homes, crowding, lack of vehicles, or group living situations.

The 226 counties that are clustered in C2 (high death rates in spring 2020 and December 2020/January 2021) are mostly populous counties in the Northeast, Washington, southeast Louisiana (including New Orleans), and the Four Corners region of Arizona and New Mexico. C2 counties experienced an early outbreak of deaths, followed by a second wave beginning in November 2020 but few deaths in summer 2020. These counties showed a strong relationship with the SVI minority and language theme, indicating a large percentage of residents who are minority or nonnative English speakers.

Cluster C4 (steady death rates beginning late summer, peaking in January) is located throughout the United States, with concentrations in the Southeast and Southwest. The counties in C4 showed a steady incidence of deaths beginning in late summer 2020 that continued through the study period. C4 counties were, on average, above the median on all SVI themes, and 3 of the 4 themes were significant in classifying counties in C4 versus C1. Specifically, the themes related to household and disability, minority and language, and housing and transportation all showed a positive association with this sustained pattern of COVID-19–related deaths. The majority (n=591, 74.4%) of these counties are located in Republican-led states.

### Limitations

The local patterns in COVID-19–related deaths suggest that local-level factors, including geographic, demographic, and social vulnerability characteristics, are related to adverse outcomes from COVID-19. There are several limitations to this research. These include the observational nature of the study, which was conducted as the pandemic continues to emerge. The retrospective, secondary use of data makes it impossible to infer causation from our model. Outbreaks and adverse outcomes changed over time as local and national governments adopted new policies and vaccines to react to the emerging pandemic. Further, the government response index is available only at the state level and is constant across all counties within a state. Using a state-level predictor to explain cluster membership at the county level could lead to an ecological fallacy.

### Conclusion

Despite limitations, this exploratory study revealed new insights into the most severe outcome of the COVID-19 pandemic. The identification of 4 distinct patterns of death incidences in 3108 US counties provides evidence of the differences in the realization of severe outcomes from the pandemic. The United States is a demographically and politically diverse nation, and it is important to understand the differences in pandemic-related outcomes across communities. By examining the relationship between county-level predictors and membership in the 4 cluster patterns, we showed that there are important demographic, political, and socioeconomic differences related to death patterns across the United States.

## Abbreviations

CDC: Centers for Disease Control and Prevention
OR: odds ratio
SVI: social vulnerability index
